# Chronic Skin Affections in the Soldiers

**Published:** 1919

**Authors:** 


**Chronic Skin Affections in the Soldiers.** By C. J. HAILPERIN, Captain M. C., U. S. ARMY. *The Journal of the American Medical Association*, November 2, 1918.

There are many types of skin affections which in ordinary civil life are of comparatively little significance. Many men go through life without suffering any great inconvenience from these troubles; however, once subjected to the physical and mental strain incident to army life, they soon become incapacitated for work and are sent to hospital for treatment.

Many of the skin diseases are of little significance from the standpoint of military service, but eczema gives considerable trouble, particularly if it affects the lower extremities. Chronic skin diseases, and particularly chronic eczema, are very resistant to treatment and usually incapacitate the men. Examining boards should inquire into the past history and be on the lookout for evidence of chronic eczema particularly. Men with chronic dermatoses should be placed under observation before they are mustered in. If they prove incurable, they should be immediately rejected.

